# Pure choriocarcinoma of the testis presenting with jaundice: a case report and review of the literature

**DOI:** 10.1186/1752-1947-6-269

**Published:** 2012-08-31

**Authors:** Mustapha Ahsaini, Fadl Tazi, Soufiane Mellas, Jallaledine Elammari, Abdelhak Khalouk, Roos Stuurman-Wieringa, Mohammed Jamal Elfassi, My Hassan Farih, Hind Elfatmi, Amal Amarti

**Affiliations:** 1 Department of urology, Hospital University Center Hassan II, Fez, 30000, Morocco; 2 Department of pathology, Hospital University Center Hassan II, Fez, 30000, Morocco; 3 Anatomy Laboratory, Faculty of Medicine and Pharmacy of Fez, Fez, 30000, Morocco; 4 Department of Urology, Reinier de Graaf Gasthuis, P.O. box 5011, 2600, Delft, GA, The Netherlands

**Keywords:** Choriocarcinoma of testis, Testicular tumor, Metastasis

## Abstract

**Introduction:**

Testicular cancer is the most common malignancy in men 15- to 35-years-old. The North American standard classification divides testicular cancers into germ cell tumors and non-germ cell tumors. The lymphatic spread of germ cell tumors usually involves the retroperitoneal lymph nodes. However, this spread to the retroperitoneum rarely involves the hepatic hilum. We describe an unusual case of metastatic choriocarcinoma of the testis that was clinically mimicked by a cholestatic jaundice. This is an unusual presentation of testicular cancer and, to the best of our knowledge, the first report of this kind in the literature.

**Case presentation:**

A 28-year-old Moroccan man presented with a four-week history of progressive obstructive jaundice, and weight loss to our emergency department. Abdominal ultrasound showed a dilatation of the biliary ducts due to pathologically enlarged lymph nodes of the hepatic hilum. A complete clinical and radiologic assessment to discover the primary tumor was negative except for pulmonary metastasis. In the laboratory findings at admission there were signs of cholestasis with an abnormal increase in the rate of testicular tumor markers (serum beta-human chorionic gonadotropin level was 11,000IU/ml), which subsequently led to the suspicion of a testicular tumor. Further evaluation included testicular palpation and ultrasound which revealed a testicular nodule. The patient underwent an inguinal orchidectomy of the right testis and histopathological examination confirmed a pure choriocarcinoma. The prognosis was poor due to lymph node involvement at the hepatic hilum. He died one month later, despite general chemotherapy.

**Conclusions:**

The clinical presentation of the disease and the rarity of this entity are two remarkable characteristics described in this case report which are rarely reported in literature.

## Introduction

In 1980, Tsuchiya *et al*. [1] described the first case of a choriocarcinoma of the testis. Since then only a few cases of choriocarcinoma have been reported. Choriocarcinoma is a germinal tumor arising from testicular cells in men or from fetal trophoblast in women. Pure testicular choriocarcinoma is a rare germ cell neoplasm, accounting for less than 3% of all the testicular neoplasms. It usually metastasizes to the lungs, liver and brain.

We present a case of pure choriocarcinoma revealed by jaundice in a young man. Despite general chemotherapy, he died due to lymph node metastasis at the hepatic hilum.

## Case presentation

A 28-year-old man who was a native of Morocco without prior medical history presented with a four-week history of progressive obstructive jaundice and weight loss to our emergency department. On physical examination he looked tired, his temperature was normal (37.5C), and he displayed conjunctival icterus. His abdominal examination was normal and a rectal examination found no tenderness or blood.

Laboratory findings at admission showed: leukocytes 12.5G/L (normal range, 4.4 to 11.3), hemoglobin 12.5g/dL (normal range, 14 to 17.5), lactic dehydrogenase 416U/L (normal range, 120 to 240), glutamic oxalacetic transaminase (GOT) 107U/L (normal, <19), glutamic pyruvic transaminase (GPT) 210U/L (normal, <23), γ-glutamyltransferase 110U/L (normal, <29), alkaline phosphatase 592U/L (normal range, 55 to 170), bilirubin 40.19μmol/L (normal range, 1.71 to 20.52), and C-reactive protein (CRP) 102mg/L (normal, <9).

**Figure 1:**
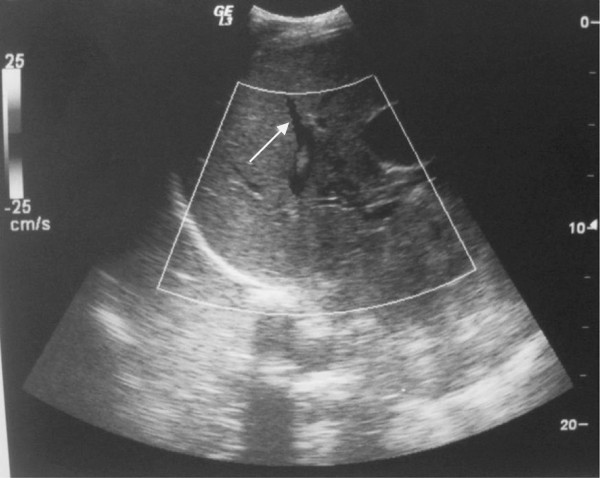
Abdominal ultrasound revealed a dilatation of the biliary ducts.

**Figure 2:**
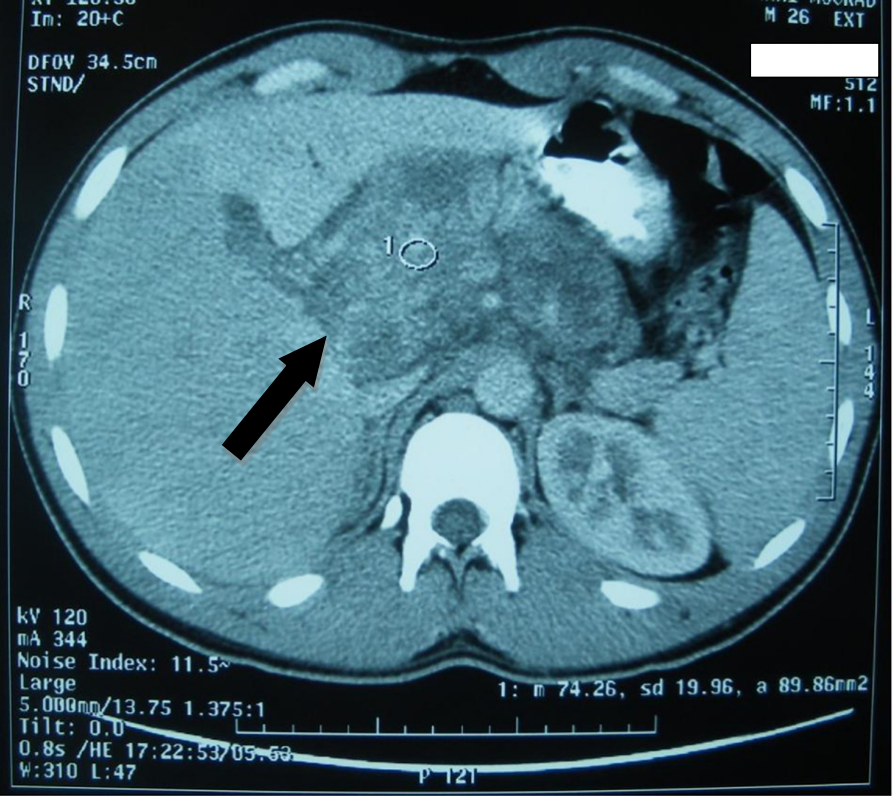
Computed tomography (CT) showed pathologically-enlarged lymph nodes of the hepatic hilium, probably partially necrotic.

Abdominal ultrasound (Figure 1) revealed a dilatation of the biliary ducts. Computed tomography (CT) (Figure 2) showed pathologically-enlarged lymph nodes of the hepatic hilum, the largest being 8.9 × 6.6cm, probably partially necrotic associated with manifold retroperitoneal lateral-aortic and interaortocaval lymph nodes. CT of the brain, chest, abdomen and pelvis showed multiple nodular lesions in both lungs, the largest being 1.2cm.

Because of his young age, we performed a complete serum analysis with testicular tumor markers. His serum beta-human chorionic gonadotrophin (HCG) level was 11,000IU/ml (normal range, 0 to 25IU/ml), serum alpha-fetal protein was 0.426IU/ml (normal < 7.2IU/ml), serum lactate dehydrogenase was 438U/L (normal range, 225 to 450U/L), carbohydrate antigen (CA19-9) and carcinoembryonic antigen (CEA) were in the normal range.

The diagnosis of testicular tumor was suspected. Further exploration included testicular palpation and ultrasound which revealed a right testicular nodule that was 2cm in its largest diameter, at the lower pole of the testis with low echogenicity and heterogeneity.

**Figure 3:**
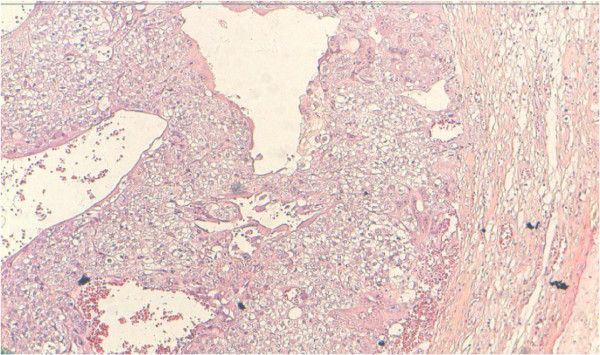
**Testicular choriocarcinoma.** A plexiform pattern with syncytiotrophoblasts covering clusters of smaller cytotrophoblasts (hematoxylin and eosin stain X 10).

**Figure 4:**
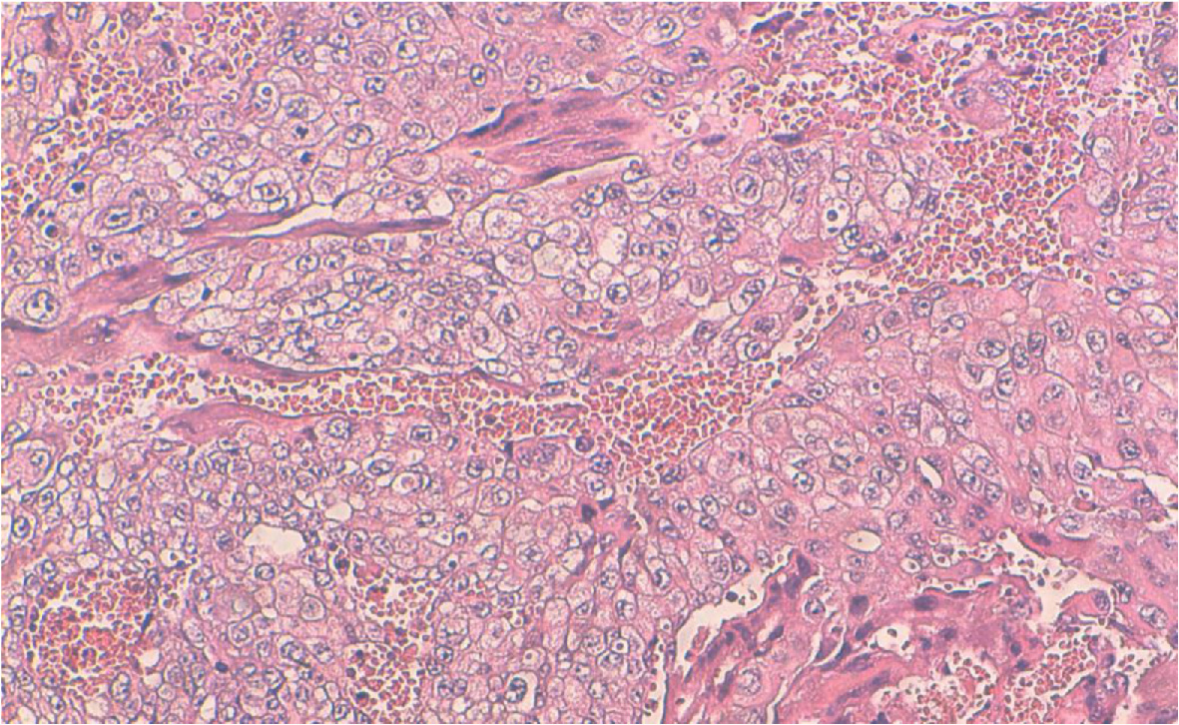
Syncytiotrophoblasts and cytotrophoblasts cells along the hemorrhagic foci (hematoxylin and eosin stain X40).

He underwent right inguinal orchidectomy and histopathological examination showed choriocarcinoma of the right testis (Figures 3, 4). Immunohistochemistry (IHC) was done after discussion with our tumor board, and the result was consistent with the diagnosis of pure choriocarcinoma. He was categorized as having a poor prognosis due to the high HCG level and the presence of lung metastasis. He was started on chemotherapy consisting of a combination of cisplatinum 20 mg/m2, etoposide 100 mg/m2 given on five consecutive days and bleomycin 30 mg on days one, eight, and 15. He died one month later due to respiratory distress.

## Discussion

Choriocarcinomas are rare, accounting for less than 1% to 3% of all the testicular neoplasms. They can also be found in combination with other germ cell tumor elements in 8% of testicular germ cell tumors [2]. Tumors are mostly found in patients in their second to fourth decade of life. Choriocarcinomas are highly malignant lesions with the potential for early, hematogenous metastases to the lung, liver, gastrointestinal tract, and brain [3,4]. Choriocarcinoma has the worst prognosis of all germ cell tumors [5]. It is a malignant growth of trophoblastic cells, which is characterized by the secretion of HCG [6]. It usually arises as gestational choriocarcinoma, from fetal trophoblasts of a previous hydatidiform mole pregnancy. In rare instances, it arises from germ cells in the testis or ovary. Choriocarcinoma is conventionally classified as pure choriocarcinoma, which is composed of only syncytiotrophoblastic and cytotrophoblastic components, or as a mixed germ cell tumor, which contains choriocarcinoma as one of the components. In men, it usually occurs as a component of testicular mixed germ cell tumors, whereas its pure form represents less than 3% of all the cases [7]. Clinical examination of the testis can be normal. This is because the primary site may be quite small, or even totally regressed, although there is widespread metastatic involvement as in our case in which the patient had a generalized metastatic disease with a small lesion in his testis. Non-seminomatous germ cell tumors of the testis usually metastasize to the retroperitoneal lymph nodes, lung, liver and brain. Metastasis to hepatic hilar lymph nodes is very rare [8,9].

Patients often have symptoms of metastasis before the primary lesion is detected. Sahraoui *et al*. [10] report in a review of the literature of 17 patients where metastases revealed the disease in seven cases (41%; lung in four cases and skin in three cases).

To the best of our knowledge, this is the first case report where jaundice is the first symptom due to larger and compressive lymph nodes of the hepatic hilum. Therefore, our patient was at first evaluated by the gastroenterology team at presentation. His young age in combination with undiagnosed abdominal or chest masses led to the measurement of tumor markers of the testis before he was referred to our institution.

On histopathological examination, the diagnosis of choriocarcinoma is based on morphologic features with the finding of cytotrophoblasts and syncytiotrophoblasts; positive staining for HCG and cytokeratin are also important. The syncytiotrophoblastic cells are usually multinucleated with deeply staining, eosinophilic to amphophilic cytoplasm, and the cytotrophoblastic cells have pale to clear cytoplasm with a single, irregularly shaped nucleus with one or two prominent nucleoli [11]. However, the demonstration of the production or the secretion of HCG is complementary to morphologic diagnosis, because other nontrophoblastic tumors (for example, lung, breast, melanoma, and gastric adenocarcinoma) can also produce detectable levels of HCG in the serum. Blood vessel invasion is commonly identified in all of the patterns [12].

The origin of the histopathological appearance is due to the strain cell of embryonal carcinomas. They grow through imitation of the trophoblastic tissue of the placenta [13].

Orchidectomy followed by chemotherapy in germ cell tumors associated with retroperitoneal lymphadenectomy is mandatory. After orchidectomy the patient underwent a protocol of four cycles of bleomycin, etoposide, and cisplatin (BEP) as recommended in the poor prognosis group with survival rates of 45% and 50% [14]. Ismaili *et al*. [15] report a good course in older men with metastatic seminoma of the testis associated with liver and renal insufficiencies who were managed successfully with carboplatin-based chemotherapy. In our case, the patient did not have any renal failure or decreased level of prothrombin, despite the presence of the hepatic metabolic disturbance. Therefore, our team did not change the standard protocol consisting of cisplatin-based chemotherapy.

The prognosis of a choriocarcinoma is worse than others due to its early hematogenous metastasis. Sahraoui *et al*. [10] reported 14 patients who had a metastasis involution after treatment of whom six died within three days to 14 months, and 11 are alive after six to 96 months.

## Conclusions

This case is rare in being pure choriocarcinoma of the testis, which accounts for less than 3% of the testicular neoplasms with an unusual clinical presentation. Investigation for primary testicular germ cell tumors is essential in young men with unidentified tumor metastasis. The detection of the testicular tumor markers may provide an important clue to the diagnosis.

## Consent

Written informed consent was obtained from the patient’s next-of-kin for publication of this case report and any accompanying images. A copy of the written consent is available for review by the Editor-in-Chief of this journal.

## Competing interests

The authors declare that they have no competing interests.

## Authors’ contribution

MA was the principal author and major contributor in writing the manuscript. MFT, SM, JA, AK and HF analyzed and interpreted the patient data and reviewed the literature. RSW, MJE, MHF and AA read and corrected the manuscript. All authors read and approved the final manuscript.

## References

[B1] Tsuchiya K Gushiken H Kamata H A case of metastatic choriocarcinoma presenting as a hemangioma-like eruption Rinsho Derma (Tokyo) 1980 22 80 81

[B2] Krag Jacobsen G Barlebo H Olsen J Schultz HP Starklint H Sogaard H Vaeth M Testicular germ cell tumours in Denmark 1976–1980. Pathology of 1058 consecutive cases Acta Radiol Oncol 1984 23 239 247 10.3109/02841868409136019 6093440

[B3] Geraghty MJ Lee FT Jr Bernsten SA Gilchrist K Pozniak MA Yandow DJ Sonography of testicular tumors and tumor-like conditions: a radiologic-pathologic correlation Crit Rev Diag Imaging 1998 39 1 63 9532420

[B4] Ulbright TM Amin MB Young RH Tumors of the Testis, Adnexa, Spermatic Cord, and Scrotum 1999 Armed Forces Institute of Pathology, Washington

[B5] Mostofi FK Price EB Mostofi FK, Price EB Tumors of the male genital system Atlas of Tumor Pathology Fascicle 8, series 2 1973 Armed Forces Institute of Pathology, Washington

[B6] Robey EL Schellhammer PF Four cases of metastases to the penis and a review of the literature J Urol 1984 132 992 994 6387189 10.1016/s0022-5347(17)49982-4

[B7] Shimizu S Nagata Y Han-yaku H Metastatic testicular choriocarcinoma of the skin. Report and review of the literature Am J Dermatopathol 1996 18 633 636 10.1097/00000372-199612000-00016 8989940

[B8] Gates O Cutaneous metastasis of malignant disease Am J Cancer 1937 30 219 228

[B9] Brownstein MH Helwig EB Metastatic tumors of the skin Cancer 1972 29 1298 1307 10.1002/1097-0142(197205)29:5<1298::AID-CNCR2820290526>3.0.CO;2-6 4336632

[B10] Sahraoui S Tahri Joueti Hassani A Ouhtatou F Acharki A Benider A Kahlain A Pure choriocarcinoma of the testis: report of a case and review of the literature Ann Urol 2001 35 125 128 10.1016/S0003-4401(01)00005-5 11355283

[B11] Eble JN Sauter G Epstein JI Sesterhenn IA World Health Organization Classification of Tumours. Pathology and Genetics of Tumours of the Urinary System and Male Genital Organs 2004 IARC Press, Lyon

[B12] Garcia RL Ghali VS Gastric choriocarcinoma and yolk sac tumor in a man: observations about its possible origin Hum Pathol 1985 16 955 958 10.1016/S0046-8177(85)80137-4 4040885

[B13] Motoyama T Sasano N Yonezawa S Matsuzaki O Kawai A Kamata Y Early stage of development in testicular choriocarcinomas Acta Pathol Jpn 1993 43 320 326 8346709 10.1111/j.1440-1827.1993.tb02574.x

[B14] Motzer RJ Nichols CJ Margolin KA Bacik J Richardson PG Vogelzang NJ Bajorin DF Lara PN Jr Einhorn L Mazumdar M Bosl GJ Phase III randomized trial of conventional-dose chemotherapy with or without high-dose chemotherapy and autologous hematopoietic stem-cell rescue as first-line treatment for patients with poor-prognosis metastatic germ cell tumors J Clin Oncol 2007 25 247 256 10.1200/JCO.2005.05.4528 17235042

[B15] Ismaili N Naciri S Afqir S Mellas N Bekkouch I Elmajjaoui S Masbah O Othmani N Flechon A Droz JP Errihani H A rare case of advanced testicular seminoma in a 78-year-old man managed successfully with carboplatin based chemotherapy: a case report Cases Journal 2008 1 357 10.1186/1757-1626-1-357 19040740 PMC2613406

